# Vaccination With Viral Vectors Expressing Chimeric Hemagglutinin, NP and M1 Antigens Protects Ferrets Against Influenza Virus Challenge

**DOI:** 10.3389/fimmu.2019.02005

**Published:** 2019-08-21

**Authors:** Meagan McMahon, Guha Asthagiri Arunkumar, Wen-Chun Liu, Daniel Stadlbauer, Randy A. Albrecht, Vincent Pavot, Mario Aramouni, Teresa Lambe, Sarah C. Gilbert, Florian Krammer

**Affiliations:** ^1^Department of Microbiology, Icahn School of Medicine at Mount Sinai, New York, NY, United States; ^2^Graduate School of Biomedical Sciences, Icahn School of Medicine at Mount Sinai, New York, NY, United States; ^3^Global Health and Emerging Pathogens Institute, Icahn School of Medicine at Mount Sinai, New York, NY, United States; ^4^Department of Biotechnology, University of Natural Resources and Life Sciences, Vienna, Austria; ^5^The Jenner Institute, University of Oxford, Oxford, United Kingdom

**Keywords:** influenza, universal influenza virus vaccine, vectored vaccine, stalk antibodies, CD8 T-cells

## Abstract

Seasonal influenza viruses cause significant morbidity and mortality in the global population every year. Although seasonal vaccination limits disease, mismatches between the circulating strain and the vaccine strain can severely impair vaccine effectiveness. Because of this, there is an urgent need for a universal vaccine that induces broad protection against drifted seasonal and emerging pandemic influenza viruses. Targeting the conserved stalk region of the influenza virus hemagglutinin (HA), the major glycoprotein on the surface of the virus, results in the production of broadly protective antibody responses. Furthermore, replication deficient viral vectors based on Chimpanzee Adenovirus Oxford 1 (ChAdOx1) and modified vaccinia Ankara (MVA) virus expressing the influenza virus internal antigens, the nucleoprotein (NP) and matrix 1 (M1) protein, can induce strong heterosubtypic influenza virus-specific T cell responses in vaccinated individuals. Here, we combine these two platforms to evaluate the efficacy of a viral vectored vaccination regimen in protecting ferrets from H3N2 influenza virus infection. We observed that viral vectored vaccines expressing both stalk-targeting, chimeric HA constructs, and the NP+M1 fusion protein, in a prime-boost regimen resulted in the production of antibodies toward group 2 HAs, the HA stalk, NP and M1, as well as in induction of influenza virus-specific—IFNγ responses. The immune response induced by this vaccination regime ultimately reduced viral titers in the respiratory tract of influenza virus infected ferrets. Overall, these results improve our understanding of vaccination platforms capable of harnessing both cellular and humoral immunity with the goal of developing a universal influenza virus vaccine.

## Introduction

Influenza A virus infections cause significant morbidity and mortality in the human population globally on an annual basis (1, 2). Current seasonal inactivated influenza virus vaccines containing influenza A virus components are most efficacious when well-matched with circulating strains. These vaccines induce strong antibody responses toward the major surface glycoprotein of the virus, the hemagglutinin (HA) (3). The influenza virus HA has two main structural components, the head and the stalk domain, and these HA-specific antibodies target the immuno-dominant head of the HA. However, the HA head undergoes substantial antigenic drift each season, rendering the previous year's vaccines ineffective at preventing infection and which forces the painstakingly cumbersome annual reformulation and re-administration of vaccines (4). Although the stalk is immuno-subdominant to the head (5), antibody responses directed toward the stalk have been shown to induce broad, heterologous protection in animal models (6). A recent study has also established anti-stalk antibody titers as independent correlate of protection in humans (7). Unfortunately, current vaccine formulations do not target the HA stalk well (8), but natural infection results in the generation of stalk antibodies in some individuals (9–12). Novel vaccine platforms are now being developed to refocus the immune response toward the immuno-subdominant HA stalk (13). One of these platforms involves sequential vaccination with chimeric HAs (cHAs), where the stalk domain of the HA remains the same, but a different head construct is used at each vaccination. Using this strategy we have previously demonstrated that vaccinating animals with the same HA stalk (but different HA heads) over consecutive immunizations, results in the production of robust antibody responses toward the stalk domain (6, 14). These stalk antibodies have the potential to be cross-reactive within and across influenza virus HA subtypes due to the high level of conservation in the stalk domain (15).

Broadly protective, heterosubtypic cellular immune responses toward the influenza virion internal antigens have also been observed in the human population (16). The internal influenza virus proteins, e.g., the nucleoprotein (NP) and matrix 1 (M1) protein, are relatively well-conserved and display limited antigenic change. T cells recognizing these antigens can be detected in most adults and are cross-reactive to influenza A virus subtypes to which the donors have not been previously exposed (17, 18). Indeed, epidemiology studies have demonstrated correlations between pre-existing T cell responses and protection from influenza disease in humans (19, 20). These studies suggest that a vaccine that primes or boosts a T cell response against highly conserved internal antigens could induce heterosubtypic protection against influenza viruses. A number of vaccine candidates have been developed to induce T cell responses (21), including replication-deficient viral vectors, such as the Chimpanzee Adenovirus Oxford 1 (ChAdOx1) and modified vaccinia Ankara (MVA) virus which express the influenza virus NP and M1 proteins and induce strong cellular immune responses toward these antigens in humans (22–25).

Given the protective efficacy that has been demonstrated by targeting the influenza virus HA stalk, NP and M1 antigens, a vaccine candidate that induces an immune response to all these antigens should be further explored. However, to augment this strategy, a strong knowledge base about the magnitude, breadth, efficacy, and protective nature of the immune response induced by immunization with cHA, NP, and M1 antigens must be built. Using ChAdOx1 and a MVA viral vectors that have been designed to co-express cHAs and the NP + M1 fusion polypeptide (cHA-NP + M1), alongside relevant monovalent controls (cHA or NP + M1) (26, 27), we vaccinated ferrets to assess the impact of the cHA-NP + M1 bivalent viral vectors on inducing both cellular and humoral immunity and inhibition of influenza virus replication in ferrets immunized with these viral vaccine candidates.

## Materials and Methods

### Ethics Statement

All animal procedures in this study were performed in accordance with the animal protocol that was reviewed and approved by the Icahn School of Medicine at Mount Sinai Institutional Animal Care and Use Committee.

### Cells and Viruses

Sf9 cells (CRL-1711, ATCC) for baculovirus rescue transfection were grown in *Trichoplusia ni* Medium-Formulation Hink (TNM-FH) insect cell medium (Gemini Bioproducts) supplemented with 10% fetal bovine serum (FBS) (Sigma) and penicillin (100 U/ml)/streptomycin (100 μg/ml) (P/S) (Gibco) solution. BTI-TN-5B1-4 (High Five) cells for protein expression were grown in serum-free SFX-insect medium (HyClone) supplemented with P/S solution. Madin Darby canine kidney (MDCK) cells, human embryonic kidney 293 (HEK293) cells, tetracycline repressor (T-Rex)-293 cell line (Thermo Fisher) and the DF-1 chicken embyo fibroblast cell line were grown in Dulbecco's Modified Eagle's Medium (DMEM) supplemented with 5% FBS and P/S solution. The influenza A virus strain, A/Wyoming/03/2003 (H3N2), was grown in 10-days-old embryonated chicken eggs (Charles River) for 48 h at 37°C. Eggs were then cooled overnight at 4°C, before the allantoic fluid was harvested the next day. Harvested allantoic fluid was centrifuged at 4,000 × g for 10 min at 4°C to remove debris. Viruses were then aliquoted and stored at −80°C.

### Recombinant Proteins

Soluble H3 [from A/Wyoming/03/2003 (H3N2)], H7 [from A/Shanghai/1/2013 (H7N9)], H10 [from A/Jiangxi-Donghu/346/2013 (H10N8)] and cH4/3 [H4 head domain from A/duck/Czechoslovakia/1956 (H4N6) and H3 stalk domain from A/Perth/16/2009 (H3N2) (28, 29)] proteins containing a T4 foldon trimerization domain and a C-terminal hexa-histidine tag for purification and NP [from A/Puerto Rico/8/1934 (H1N1)] and M1 [from A/Puerto Rico/8/1934 (H1N1)] with N-terminal hexa-histidine tags were generated using the baculovirus expression system as previously described (30, 31).

### Viral Vectors

For the ChAdOx1 viral vectors, antigens were inserted at the E1 locus of the ChAdOx1 genome, using the CMV promoter to drive antigen expression. Monovalent vaccines expressed either cH14/3 consisting of the head of H14 [from A/mallard/Guryev/263/1982 (H14N5)] and the H3 stalk [A/Perth/16/2009 (H3N2)] (29), or NP+M1 consisting of the NP and M1 sequences from A/Panama/2007/1999 joined by a 7-amino acid linker sequence (26). The bivalent vaccine expressed NP+M1 fused to cH14/3 via a 2A ribosome skipping sequence to allow expression of the two antigens as individual proteins. For MVA viral vectors, antigen-coding sequences were inserted at the F11 site (32) using the endogenous F11 promoter to drive expression of NP+M1 or the mH5 promoter (33) to drive expression of cH15/3, consisting of the head of H15 [from A/shearwater/West Australia/2576/1979 (H15N9)] and the H3 stalk [A/Perth/16/2009 (H3N2)] (29). The bivalent MVA vector contained the F11 promoter expressing NP+M1 followed immediately by the mH5 promoter expressing cH15/3, all within the F11 insertion site. The Viral Vector Core Facility at the Jenner Institute produced all vectored vaccines. ChAdOx1 vaccines were produced using the T-Rex-293 cell line (Thermo Fisher) and purified by cesium chloride density gradient centrifugation. MVA vaccines were produced using the DF-1 cell line (34) and purified using 36% sucrose cushion centrifugation.

### Ferret Experiments

**Figure 1:**
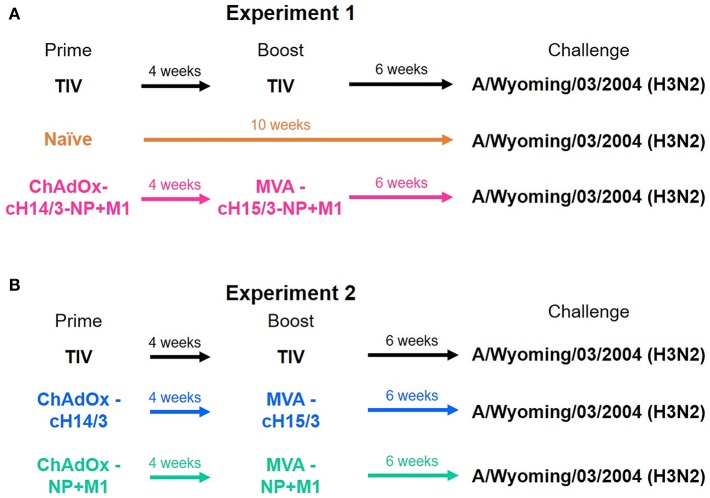
Ferret vaccination regime. In the first experiment ferrets were either prime-boosted with the virus-matched TIV, primed with ChAdOx1-cH14/3-NP + M1 and boosted 4 weeks later with MVA-cH15/3-NP + M1 or were naïve **(A)**. In the second experiment ferrets were either prime-boosted with the virus-matched TIV, primed with ChAdOx1-cH14/3 and boosted 4 weeks later with MVA-cH15/3 or primed with ChAdOx1-NP + M1 and boosted with MVA-NP + M1 **(B)**. The study was split into two experiments due to space limitations. The TIV groups served as bridging groups.

Four-months-old castrated male Fitch ferrets were purchased from Triple F Farms. Ferrets were confirmed seronegative to influenza A virus by hemagglutination inhibition assay as described below. Ferrets were randomly assigned to different vaccination groups over two experiments. Figure 1 summarizes the prime/boost vaccinations administered to each group. The study was split into two experiments (Experiment 1, Experiment 2) due to space limitations in the animal facility. To be able to compare the two studies a trivalent influenza vaccine (TIV) vaccinated group was included in both experiments. Ferrets that were primed intramuscularly (IM) with ChAdOx1 viral vectors received 5 × 10^8^ infectious units (IU) and were subsequently boosted IM 4 weeks later with 1 × 10^8^ plaque forming units (PFU) of MVA. Ferrets that were primed and boosted with the infection matched (A/Wyoming/03/2003) 2004–2005 influenza season TIV (Fluzone, BEI Resources) received the full human dose twice (2 × 15 μg of the matched HA). In the first experiment ferrets were either primed and boosted with the virus-matched TIV, primed with ChAdOx1-cH14/3-NP + M1 and boosted with MVA-cH15/3-NP + M1 or were mock immunized (naïve). In the second experiment ferrets were either primed and boosted with the virus-matched TIV, primed with ChAdOx1-cH14/3 and boosted with MVA-cH15/3 or primed with ChAdOx1-NP + M1 and boosted with MVA-NP + M1.

Six weeks post-boost, all animals were anesthetized and challenged intranasally (IN) with 10^6^ PFU of A/Wyoming/03/2003 H3N2 virus in 1 mL of phosphate buffered saline (PBS). Nasal wash and oropharyngeal swab samples were collected from anesthetized ferrets on days 1 and 3 after challenge. On day 4 post-challenge, under anesthesia, ferrets were euthanized by intra-cardiac injection of SleepAway euthanasia solution (Fort Dodge, IA) and tissues (nasal turbinate, olfactory bulb, trachea and lung) were collected from each individual ferret and were homogenized using a Bead Blaster 24, oscillating at 6 m/s for 30 s. The homogenate was then centrifuged, and the supernatant was aliquoted and stored at −80°C prior to quantifying viral titers by plaque assay. Spleen and mediastinal lymph nodes (MLNs) were also collected at day 4 post-challenge to detect cellular immune responses by enzyme linked ImmunoSpot (ELISpot) and flow cytometric intracellular cytokine staining (ICS) assays. Serum was collected prior to viral challenge for the determination of antibody responses by ELISA.

### Virus Titration

Virus titers were determined by plaque assay on MDCK cell monolayers as previously described (35). Briefly, virus stocks and experimental samples were diluted 10-fold in infection medium and allowed to absorb to cells for 1 h, with shaking every 15 min. After 1 h, an agarose overlay containing 0.64% agarose (Oxoid), 1 × minimum essential medium (MEM) [10% 10 X MEM (Gibco), 2 mM L-glutamine (Gibco), 0.1% sodium bicarbonate (Gibco), 10 mM 4-HEPES (2-hydroxyethyl)-1-piperazineethanesulfonic acid (Gibco), 100 U/mL penicillin-100 μg/ML streptomycin (Gibco), and 0.2% bovine serum albumin (BSA)] and 1 μg/mL tosyl phenylalanyl chloromethyl ketone (TPCK) -treated trypsin was added to the cells. The cells were then incubated for 48 h at 37°C and visible plaques were counted after fixing with 3.7% formalin and visualization with a crystal violet counterstain (Sigma-Aldrich). Virus titers of nasal wash and oropharyngeal swab samples are presented as PFU/mL and respiratory tract virus titers are presented as PFU/gram of tissue.

### Enzyme-linked Immunosorbent Assay (ELISA)

ELISA plates (Immunolon 4HBX; Thermo Scientific) were coated with 2 μg/mL of recombinant protein (50 μL per well) in coating buffer (KPL coating solution, Sera Care) at 4°C overnight. The following day, the plates were washed 3 times with PBS containing 0.1% Tween-20 (PBS-T) and blocked in blocking solution (3% goat serum, 0.5% milk in PBS-T) for 1 h at room temperature (RT). After blocking, pre-diluted naïve and pre-challenge serum was added to the first well to a final concentration of 1:100 in blocking solution. Serum was then serially diluted and incubated at RT for 2 h. Plates were then washed three times with PBS-T before adding goat anti-ferret IgG horseradish peroxidase (Abcam) at a concentration of 1:5,000 in blocking solution for 1 h at RT. Following a final PBS-T wash with shaking, the *O*-phenylenediamine dihydrochloride (OPD) substrate (SigmaFast OPD; Sigma) was added to each well. After 10 min of incubation at RT, the reaction was stopped by adding 50 μl of 3 M HCl (Fisher Scientific) to the mixture. The optical density (OD) was measured at 490 nm on a Synergy 4 plate reader (BioTek). A cutoff value of the average of the OD values of blank wells plus 3 standard deviations was established for each plate and used for calculating the area under the curve (AUC), which was the readout for this assay. The limit of detection of the assay was an AUC below 100, samples that did not reach this titer were assigned a value of 1:50 for graphing purposes.

### Hemagglutination Inhibition (HI)

To remove non-specific inhibitors of hemagglutination, pre-challenge serum samples were treated with receptor-destroying enzyme (RDE; Denka Seiken, Tokyo, Japan) overnight at 37°C. To stop RDE treatment, sodium citrate (2.5%) was added and serum was incubated at 56°C for 1 h. The inactivated serum samples (dilution of 1:10) were serially diluted 2-fold in PBS in 96 well V-bottom plates. Four hemagglutinin units/well of A/Wyoming/03/2003 was added to each well and the plates were allowed to incubate at room temperature for 30 min. After the virus/serum, incubation, 50 μL of 0.5% turkey red blood cells (Lampire), diluted in PBS, was added to each well. The plates were kept at 4°C for 30–45 min and scanned, and the results were analyzed in Microsoft Excel and GraphPad Prism 7. Titers of 1:5 were assigned to HI-negative subjects to facilitate data analysis and data representation.

### Microneutralization Assay

RDE inactivated serum samples (dilution of 1:10) were serially diluted 2-fold in 1xMEM, supplemented with TPCK-treated trypsin at a concentration of 1 μg/ml, in 96-well cell culture plates (Sigma). The A/Wyoming/03/2003 virus was diluted to a concentration of 100 50% cell culture infectious doses (TCID_50_) in infection medium. Sixty microliters of serially diluted serum was incubated with 60 μl of virus dilution for 1 h at room temperature on a shaker. MDCK cells were washed once with 220 μl of PBS, and 100 μl of the virus-serum mixture was added to MDCK cells. The cells were incubated for 48 h at 33°C. After 48 h, the readout was performed by the means of a hemagglutination assay as described above.

### Interferon-γ (IFNγ) ELISpot

Spleens and MLNs were aseptically removed from vaccinated ferrets at 4 days after the IN challenge. Tissues were then pushed through a 70-μM filter (Foxx Life Sciences) to create single-cell suspensions in alpha MEM (Sigma-Aldrich) medium, supplemented with 5% FBS and P/S solution. Cells were resuspended at 2.5 × 10^6^ cells/mL and 50 μL of the cell suspension was added to an ELISpot multi-screen 96-well filter plate (Merck Millipore). These plates had been previously coated with the IFNγ ferret specific mAb overnight at 4°C at 15 μg/mL (Mabtech). Peptide pools spanning the length of the NP+M1 fusion polypeptides were then added to stimulate cells at a final concentration of 1 μg/mL (36). Medium alone was used as a negative control. The plates were incubated for 40 h at 37°C and then washed 5 times with PBS. After washing, the biotinylated detection antibody was added (Mabtech), and the plates were left for 2 h at RT. The plates were then washed 5 times with PBS and the streptavidin-alkaline phosphatase was added for 1 h at RT (Mabtech). The plates were washed as above and the IFNγ secreting cells were detected using the alkaline phosphatase conjugate substrate kit (Bio-Rad), as per the manufacturer's instructions. Results are expressed as spot forming units (SFU) per million cells, calculated by subtracting the mean negative control response from the mean of the response to the peptide arrays.

### ICS Assay

Influenza virus-specific T cell responses were assessed using the ICS assay. Briefly, single cell suspensions from the nasal wash, spleen and MLNs were stimulated in a 96-well round bottom plate (Falcon) with peptides spanning the NP+M1 fusion protein in the presence of Brefeldin-A (BioLegend) at 37°C in alpha MEM. Five hours later splenocytes were stained for the presence of anti-CD8a eFluor450 labeled mAb (OKT-8, Thermo Fisher Scientific) and were then permeabilized in Perm-wash (BioLegend) followed by staining with an anti-IFNγ fluorescein isothiocyanate (FITC) labeled mAb (CC302, Abcam) as previously described (37). Data was acquired on a BD-LSRII flow cytometer and analyzed using FlowJo software.

### Statistics

The data are presented as individual replicates with the group with the average as line; *n* represents the number of ferrets per experiment. Statistical differences between groups were determined by one-way analysis of variance (ANOVA), followed by a Bonferroni multiple-comparison test. One-way ANOVA was selected as the groups were normally distributed and there was one independent variable. All statistical analyses were performed using GraphPad Prism 7 for Windows. In all cases, probability levels of 0.05 or less (^*^p ≤ 0.05) were indicative of statistical significance (^**^p ≤ 0.01, ^***^p ≤ 0.001).

## Results

### Vaccination With Viral Vectors Expressing cHA and NP + M1 Leads to Control of Viral Infection in Ferrets

**Figure 2:**
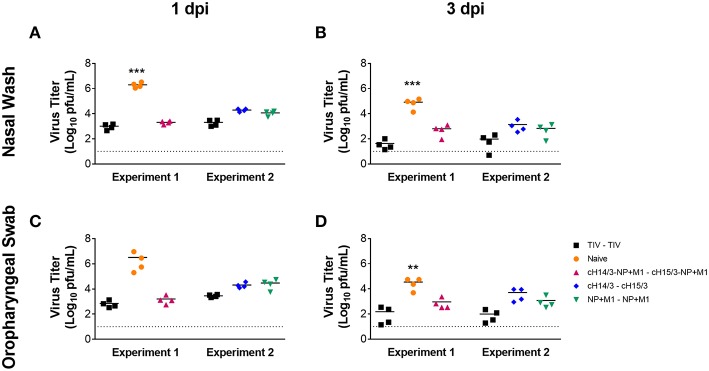
Nasal wash and oropharyngeal titres at days 1 and 3 post-challenge. Ferrets were prime-boosted with TIV or viral vectors (ChAdOx1, then MVA) expressing influenza virus antigens (cHA-NP + M1, cHA, or NP + M1). Six weeks after the boost, ferrets were challenged IN with 10^6^ PFU of A/Wyoming/03/2003 (H3N2) virus and nasal washes were collected at days 1 **(A)** and 3 **(B)** post-challenge to detect viral titres via plaque assay. Oropharyngeal swabs were also collected at days 1 **(C)** and 3 **(D)** post-challenge to determine viral titres. The data are presented as individual replicates and the mean for each group is presented as line. *n* = 4 ferrets/group. ^**^p ≤ 0.01, ^***^p ≤ 0.001 when naïve controls were compared to individual vaccination groups as determined by one-way analysis of variance, followed by a Bonferroni multiple-comparison test.

**Figure 3:**
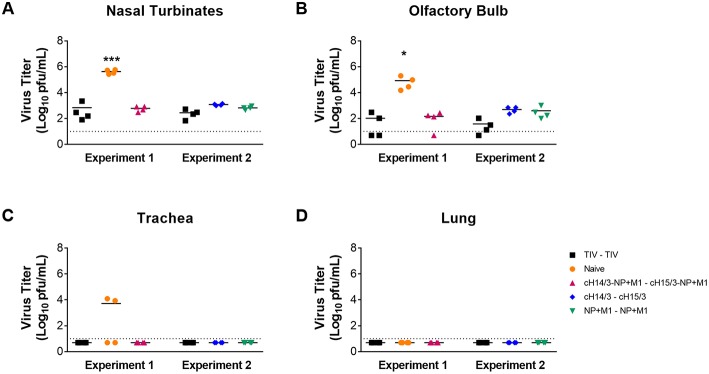
Viral titres in the respiratory tract at day 4 post-challenge. Ferrets were prime-boosted with TIV or viral vectors (ChAdOx1 and MVA) expressing influenza antigens (cHA-NP + M1, cHA, or NP + M1). Six weeks after the boost, ferrets were challenged IN with 10^6^ PFU of A/Wyoming/03/2003 (H3N2) virus and at 4 days post-challenge ferrets were euthanized and respiratory tissues were taken to detect virus titres in the nasal turbinates **(A)**, olfactory bulb **(B)**, trachea **(C)**, and lung **(D)**. The data are presented as individual replicates and the mean for each group is presented as line. *n* = 4 ferrets/group. ^*^p ≤ 0.05, ^***^p ≤ 0.001 when naïve controls were compared to individual vaccination groups as determined by one-way analysis of variance, followed by a Bonferroni multiple-comparison test.

To determine if vaccination with viral vectors could protect ferrets from influenza virus infection (Figure 1), ferrets were either IM primed and boosted with the virus-matched TIV (human dose, given twice), or primed with ChAdOx1-cH14/3-NP + M1 and boosted with MVA-cH15/3-NP + M1, or primed with ChAdOx1-cH14/3 and boosted with MVA-cH15/3, or primed with ChAdOx1-NP + M1 and boosted with MVA-NP + M1 or were mock-immunized (naïve). Of note, due to space constraints the study was split into two experiments (Experiment 1, Experiment 2) which were performed at identical conditions. A trivalent influenza vaccine (TIV) group was included in both experiments to allow comparison between them. To determine if vaccination with viral vectors could protect ferrets from influenza virus infection (Figure 1), we IN infected ferrets with 10^6^ PFU of A/Wyoming/03/2003 H3N2 virus 6 weeks after the booster vaccination and compared viral titers in the nasal washes and oropharyngeal swabs at 1- and 3-days post-infection (dpi). Our results show that vaccination with all viral vectors (NP + M1, cHA or cHA-NP + M1) and the virus-matched TIV reduced viral titers in the nasal washes at both days 1 (Figure 2A) and 3 (Figure 2B) post-challenge infection when compared to naïve controls. These findings were repeated in the oropharyngeal swabs at both days 1 (Figure 2C) and 3 (Figure 2D) post-challenge. There was no significant difference between the vaccination groups. However, ferrets vaccinated with the bivalent cHA-NP+M1 vaccines had reduced virus titers in comparison with the cHA and NP + M1 groups in the 1 dpi nasal washes and oropharyngeal swabs (trend, not significant).

To compare differences in viral titers in respiratory tract tissues (nasal turbinates, olfactory bulb, trachea, and lung), ferrets were euthanized at day 4 post-challenge and differences in virus titers in the respiratory tract tissue were assessed via plaque assay. We found that all viral vector vaccination regimes (NP + M1, cHA, and cHA-NP + M1) and the virus-matched TIV had significantly reduced viral titers in the nasal turbinates (Figure 3A) and olfactory bulb (Figure 3B) when compared to naïve controls. When assessing viral titers in the trachea we detected no virus replication in either TIV, cHA-NP + M1, cHA, or NP + M1 vaccinated ferrets, however virus was found in 2 out of 4 of the naïve controls (Figure 3C). Viral titers were below the limit of detection in the lungs of all ferrets (Figure 3D), which is not surprising given the fact that more recent H3N2 isolates have been reported to be incapable of replicating in ferret lungs (38). These results indicate that vaccination with the bivalent cHA-NP+M1 viral vectors and the monovalent cHA and NP + M1 viral vectors significantly reduced influenza viral titers in comparison with naïve control ferrets.

### Vaccination With Viral Vectors Expressing cHA Induces Broadly Reactive Antibody Responses Toward Influenza Antigens

**Figure 4:**
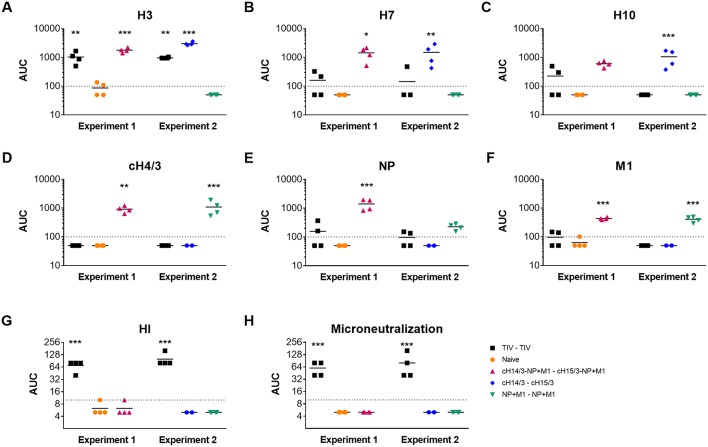
Antibody responses toward influenza virus antigens in the pre-challenge serum of vaccinated ferrets. Six weeks after the booster vaccination, pre-challenge serum was collected from TIV vaccinated, viral vector vaccinated, and naïve ferrets and IgG antibody responses toward influenza virus antigens were assessed via ELISA. Shown are antibody responses toward the H3 **(A)**, H7 **(B)**, H10 **(C)**, cH4/3 **(D)**, NP **(E)**, and M1 **(F)** recombinant proteins. HI **(G)** and microneutralization titres **(H)** were also assessed. The data are presented as individual replicates and the mean for each group is presented as line. *n* = 4 ferrets/group. ^*^p ≤ 0.05, ^**^p ≤ 0.01, ^***^p < 0.005 when naïve controls were compared to individual vaccination groups as determined by one-way analysis of variance, followed by a Bonferroni multiple-comparison test.

After measuring decreased viral titers in the viral vector-vaccinated ferrets, we wanted to measure the induced antibody responses toward the influenza virus antigens incorporated in the vaccines. We collected pre-challenge serum from ferrets and measured their antibody responses toward the recombinant antigens using an ELISA. Here, we found that ferrets vaccinated with TIV only induced robust immune responses toward the matched H3 HA (Figure 4A). Ferrets vaccinated with the bivalent and monovalent viral vectors expressing cHAs had a greater breadth of antibody response. Here, we assessed reactivity toward group 2 HAs that cause sporadic zoonotic infections (H7, H10). Antibodies against H7 and H10 HAs were detected in both the cHA or cHA-NP + M1 groups (Figures 4B,C). As expected, these antibodies were not detected in the monovalent NP + M1 viral vector vaccinated group or the naïve group. We also detected H3 anti-HA stalk antibodies in ferrets vaccinated with the bivalent and monovalent viral vectors expressing cHAs, that were not detectable in mock, TIV or NP + M1 viral vector vaccinated ferrets (Figure 4D). In addition, we assessed antibody responses toward NP and M1 recombinant proteins and found that ferrets vaccinated with viral vectors containing the NP + M1 had antibody responses toward these antigens (Figures 4E,F). These antibody responses were not observed in the TIV or the cHA vector vaccination groups or the naïve controls, although the TIV may contain some of these antigens at a non-standardized concentration (3, 39). We also measured HI and microneutralization titers, but only detected titers in the TIV vaccinated groups (Figures 4G,H). In support of other studies, our results demonstrate that vectored vaccines expressing influenza antigens can induce antibody responses toward these antigens.

### Vaccination With Viral Vectors Expressing Influenza Virus Antigens Induces IFN-γ Cellular Responses in Ferrets

**Figure 5:**
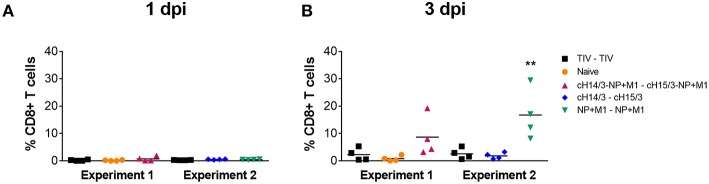
Influenza virus specific T cells in the nasal washes. TIV vaccinated, viral vector vaccinated (cHA-NP + M1, cHA, or NP + M1), and naïve ferrets were challenged IN with 10^6^ PFU of A/Wyoming/03/2003 (H3N2) virus and nasal washes were collected at days 1 and 3 post-challenge. Cells were isolated from these nasal washes and stimulated with peptide arrays spanning the NP + M1 fusion protein. Following stimulation, cells were stained with anti-CD8 and -IFN-γ antibodies and influenza virus-specific IFN-γ^+^CD8^+^ T cells were measured at days 1 **(A)** and 3 **(B)** post-challenge. The data are presented as individual replicates and the mean for each group is presented as line. *n* = 4 ferrets/group. ^**^p < 0.01 when naïve controls were compared to individual vaccination groups as determined by one-way analysis of variance, followed by a Bonferroni multiple-comparison test.

After discovering the robust antibody responses in viral vector vaccinated ferrets toward HAs, NP, and M1 (Figure 4), we next wanted to determine if cellular responses could also contribute to differences in virus titers, given that ferrets vaccinated with viral vectors containing the NP + M1 fusion polypeptide also had reduced virus titers (Figures 2, 3). We therefore measured CD8^+^ T cells in the nasal cavity at early time points post-infection. To do this we collected the cellular component from the nasal washes taken at days 1 and 3 post-challenge and stimulated these cells with peptide pools spanning the NP + M1 fusion polypeptide (36). Using the intracellular cytokine staining (ICS) assay, we found no detectable differences in the percentage of influenza virus NP + M1 specific CD8^+^ (Figure 5A) T cells at day 1 post-challenge between the separate groups. However, an increased number of influenza virus NP + M1 specific CD8^+^ T cells were found in the nasal cavity at day 3 post-challenge in ferrets who received viral vectors containing the NP + M1 fusion protein (Figure 5B).

**Figure 6:**
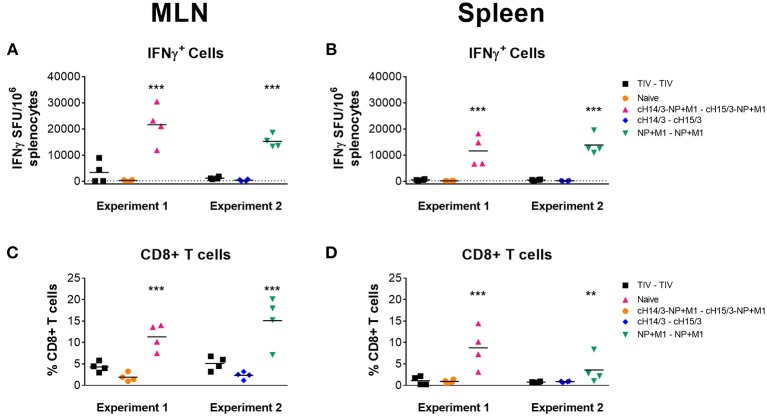
IFNγ cellular responses in the MLNs and spleen. TIV vaccinated, viral vector vaccinated (cHA-NP + M1, cHA, or NP + M1), and naïve ferrets were challenged IN with 10^6^ PFU of A/Wyoming/03/2003 (H3N2) virus and at 4 days post-challenge, ferrets were euthanized, and spleens and MLNs were removed to enumerate influenza virus-specific cellular responses. Splenocytes and MLN cells were stimulated with peptide arrays spanning the NP + M1 fusion protein and influenza virus-specific IFN-γ expressing cells were detected in the MLNs **(A)** and spleens **(B)** by ELISpot. The ICS assay was also used to detect influenza virus-specific IFNγ^+^CD8^+^ T cells in the MLN **(C)** and spleen **(D)**. The data are presented as individual replicates and the mean for each group is presented as line. *n* = 4 ferrets/group. ^**^p ≤ 0.01, ^***^p ≤ 0.001 when naïve controls were compared to individual vaccination groups as determined by one-way analysis of variance, followed by a Bonferroni multiple-comparison test.

We further expanded our T cell analysis by measuring IFN-γ responses in the spleen and MLNs at day 4 post-challenge. Here, we stimulated cells with peptides spanning the NP + M1 fusion polypeptide and found increased numbers of influenza virus specific IFN-γ^+^ T cells in the MLNs (Figure 6A) and spleen (Figure 6B) of ferrets who had been vaccinated with viral vectors containing the NP + M1 fusion protein, using the ELISpot assay. When we assessed influenza virus specific CD8^+^ T cell responses via the ICS assay, we identified increased numbers of these cells in the MLNs (Figure 6C) and spleen (Figure 6D) of ferrets that received vectored vaccines containing the NP + M1 fusion protein, alone or with cHA.

## Discussion

The aim of this study was to combine two of the leading vaccination approaches that have been individually shown to induce broadly cross-reactive either anti-influenza virus humoral or cellular immune responses. In the first approach, sequential exposure to cHAs (with a variable head but the same stalk) refocuses the antibody response toward the immuno-subdominant stalk, inducing robust anti-stalk antibodies (6, 40–43). In the second approach, replication-deficient viral vectors expressing the internal NP and M1 antigens induce long-lived, heterosubtypic T cell responses (22, 23). In this study we show that viral vectors expressing cHAs, NP and M1 influenza antigens can induce both broad anti-HA antibodies and IFN-γ cellular immune responses in ferrets that are protective against influenza virus infection. Indeed, ferrets that were vaccinated with either the cHA monovalent or the cHA-NP + M1 bivalent viral vector had reduced viral titers in comparison to naïve controls (Figures 2, 3) which can likely be attributed to robust antibody responses. These antibody responses may be capable of protecting against other group 2 influenza viruses given that we observed increased antibody responses to other group 2 HAs following vaccination (Figure 4). Additionally, ferrets that were vaccinated with viral vectors expressing the NP + M1 monovalent or cHA-NP + M1 bivalent antigens had detectable T cells in the nasal cavity at day 3 post-challenge (Figure 5) and significantly increased influenza virus-specific T cells in the lymphoid tissue (Figure 6). This might have led to reduced viral titers in these vaccinated ferrets upon influenza virus infection (Figures 2, 3). While not assessed, it is also possible that T cell responses against the HA could have contributed to protection in the cHA and cHA-NP + M1 vaccine groups. Of note, while no heterosubtypic challenge was performed it is important to state that both the H3 stalk of the cHAs (from A/Perth/16/09) and the NP and M1 (from A/Panama/2007/1999) are mismatched to the challenge strain (A/Wyoming/03/2003).

In mouse studies using these same viral vectors, we found that animals vaccinated with the monovalent viral vectors were protected from lethality, but significant levels of weight loss were observed (44). In contrast, vaccinating mice with the bivalent viral vectors fully protected mice from infection and limited weight loss was observed (44). However, this was not observed in our study in ferrets in which we saw equivalent protection afforded by cHA alone, NP + M1 alone or bivalent vaccines. These differences may be attributed to the different readouts used for assessing protection. In the mouse model, we used lethal challenge doses and inferred protection via weight loss and survival. For this study we used a sub-lethal dose of virus (H3N2 viruses do typically not cause death in ferrets) and we attributed protection by measuring differences in virus titers which may not allow us to detect minute differences. Antibody responses following cHA vaccination were broadly responsive to group 2 HAs and targeted the HA stalk domain and this suggests that they might protect ferrets from heterologous challenge (45, 46). The induction of such antibodies in mice largely protected animals from lethal challenge with A/Shanghai/1/2013 (H7N9) and A/Jiangxi-Donghu/346/2013 (H10N8) viruses (44). We also assessed antibody responses toward NP and M1 recombinant proteins and observed increased antibodies in the ferrets vaccinated with viral vectors expressing the NP+M1 fusion protein. The sequences for the proteins used in these assays were derived from the A/Puerto Rico/8/1934 (H1N1) (PR8) influenza virus. However, the NP + M1 fusion protein sequence is from the A/Panama/2007/1999 (H3N2) influenza virus strain. Although we detected antibody responses in the ferrets vaccinated with the viral vectors expressing the NP + M1 fusion polypeptide toward the PR8 derived internal proteins, it is unlikely that these antibodies afford robust protection against influenza virus infection and protection is likely mostly mediated by influenza specific T cells. Given the high conservation of NP and M1, we believe that vaccination with the NP + M1 fusion protein will lead to heterosubtypic immunity against group 1 and group 2 influenza viruses elicited by virus-specific T cells. In addition, NP and M1 antibody responses may be beneficial to a certain degree given that NP antibodies have been shown to have some anti-viral activity (47, 48).

In summary, our data suggests that very different mechanisms afford protection in the different vaccination regimens. TIV induced neutralizing and HI active antibodies but very little cross-reactivity and basically no T cell responses, suggesting that the main mechanism in this group is virus neutralization. This led to a drastic reduction in virus replication and is in line with results from other groups testing matched vaccines in H3N2 ferret challenge models (49, 50). Animals vaccinated with viral vectors expressing NP and M1 were most likely protected by T cell responses targeting these proteins, potentially with a minor role in anti-NP and anti-M1 antibodies as outlined above. Animals receiving viral vectors expressing cHAs only were likely protected by anti-HA stalk antibodies. No strong neutralizing activity was detected in these animals and it is likely that protection was mostly based on Fc-Fc receptor interactions (contribution of low neutralizing activity cannot be ruled out). We attempted to measure this activity using commercial human antibody dependent cellular cytotoxicity (ADCC) and antibody dependent cell-mediated phagocytosis (ADCP) reporter assays, but could not detect any activity, likely due to the low sensitivity of the species mismatched reagents. We successfully used the human ADCC reporter assay in the past in ferrets, but these animals were vaccinated three times with cHAs and despite their higher antibody titers showed only very low activity in the assay (46). This highlights the need for the development of more reagents for the ferret model (51). As mentioned above, combining cHA and NP+M1 strategies did not improve protection significantly, although a trend to lower nasal wash viral titers was observed early during the challenge. It is possible that both Fc-Fc receptor depending effector functions and T cell responses are similar in function and timing and therefore produce no additive or synergistic effect. Differences between the various vector vaccinated groups may be better observable following challenge with more divergent influenza viruses, such as an H7N9 influenza virus isolate (52). Importantly, the NP + M1, the cHA, and the cHA-NP + M1 vectored approach resulted in protection that was comparable to protection induced by matched TIV.

Inclusion of both the cHA and the NP + M1 fusion protein in the same viral vector could have limited the ability for antigen processing, and hence reduce immune response to one or both antigens. However, we have identified no obvious dampening of the immune response between the monovalent and bivalent viral vectors in both vaccinated mice and ferrets in our studies. Indeed, we found antibody responses toward the H3, H7, H10, and cH4/3 HA in both the cHA and cHA-NP + M1 viral vector vaccinated groups. Antibodies against NP and M1 were even slightly higher in the group vaccinated with the cHA-NP+M1 regimen than in the animals vaccinated with NP+M1 vectors. Additionally, comparable IFN-γ responses were observed in the NP + M1 and cHA-NP + M1 vector vaccinated groups upon stimulation with peptides spanning the NP + M1 fusion protein in mice and ferrets. This lack of dampening and presence of cross-reactive, broadly cytotoxic responses would be relevant in a pandemic context, during vaccine failure or for longitudinal responses.

These highly encouraging data suggest that the viral vectors used in this study might produce similar results in clinical studies. The used viral vectors can be produced at high titers and at large scale. Phase I studies of vaccines expressing cHA are underway (clinicaltrials.gov NCT03300050 and NCT03275389), and multiple clinical studies have been conducted with ChAdOx1 and MVA expressing NP + M1 (22–24, 26). It is anticipated that vaccination with the bivalent vectors described here would most likely induce both robust anti-HA stalk antibodies and long-lived T cell responses in humans.

It is widely acknowledged that vaccines targeting multiple conserved influenza virus antigens offer a novel approach for the development of a universal influenza virus vaccine. Our data show, and support, the development of a vaccination regime that induces an immune response to multiple conserved influenza virus antigens that can protect from influenza virus infection.

## Data Availability

All datasets generated for this study are included in the manuscript/supplementary files.

## Author Contributions

MM, GA, W-CL, DS, and RA performed the animal experiments. TL, VP, and MA constructed the viral vectors. MM, TL, SG, and FK designed the experiments, analyzed the data, and wrote the manuscript.

### Conflict of Interest Statement

The Icahn School of Medicine at Mount Sinai has filed patent applications regarding influenza virus vaccines with FK being an inventor. SG is an inventor on patents covering ChAdOx1 and MVA-NP + M1, filed and owned by the University of Oxford, and is a co-founder of and consultant to Vaccitech, a University of Oxford spin-out company which is undertaking advanced clinical development of viral vectored influenza vaccines. The remaining authors declare that the research was conducted in the absence of any commercial or financial relationships that could be construed as a potential conflict of interest.
